# Blockage of cuproplasia inhibits pancreatic tumour-associated neutrophils infiltration through TRAF6/STAT3/CCL2 pathway

**DOI:** 10.1038/s41416-026-03371-8

**Published:** 2026-04-14

**Authors:** Ruiman Geng, Huawei Cai, Xuxu Ji, Xiaoding Shen, Ziyao Wang, Zhao Li, Ruomeng Liu, Zhengkun Zhang, Dingxue Wang, Zhaoru Yin, Jiaqiong Zou, Rong Guo, Panpan Dai, Zhiyou Quan, Lihong Chen, Nengwen Ke, Ji Liu

**Affiliations:** 1 Department of Biochemistry and Molecular Biology, West China School of Basic Medical Sciences & Forensic Medicine, Sichuan University, Chengdu, China; 2 Department of Laboratory Medicine, The First Affiliated Hospital of Xi’an Medical University, Xi’an, China; 3 Laboratory of Clinical Nuclear Medicine and Department of Nuclear Medicine, West China Hospital, Sichuan University, Chengdu, China; 4 Sichuan Provincial Engineering Research Center of Radiopharmaceutical Clinical Translation, Sichuan University, Chengdu, China; 5 Department of Pancreatic Surgery, West China Hospital, Sichuan University, Chengdu, China

**Keywords:** Cell signalling, Cancer metabolism, Tumour immunology, Chemokines

## Abstract

**Background:**

Pancreatic ductal adenocarcinoma (PDAC) is a highly aggressive cancer with a poor prognosis and is easy to developing drug resistance to conventional therapies due to its distinctive tumour microenvironment. Recent advancements have brought attention to the aberrant copper metabolism in this malignancy, but the influence of intracellular cuproplasia and balance on the tumoral immune microenvironment is still uncertain.

**Methods:**

We analysed copper concentrations and CTR1 expression in PDAC tissues and cell lines. Spatial transcriptomics was employed to delineate the relationship between CTR1 overexpression and tumour-associated neutrophils (TANs) infiltration. Chemokine arrays and molecular assays were used to identify key signalling pathways involved. Functional experiments, including CTR1 knockdown and TRAF6 overexpression, were conducted to assess its impact on neutrophil infiltration and therapeutic synergy with gemcitabine.

**Results:**

We identified that CTR1 overexpression drives intracellular copper overaccumulation, activating TRAF6-dependent phosphorylation of JAK/STAT3. Phosphorylated STAT3 transcriptionally upregulates the chemokine CCL2, fostering CCR2-mediated TANs infiltration, which correlates with poor prognosis. Crucially, single-cell RNA sequencing revealed CTR1 knockdown suppresses a pro-metastatic TAN subpopulation (TAN-2) and dramatically reduces TANs recruitment in orthotopic tumour models. This copper-targeted intervention concurrently enhances cytotoxic CD8^+^ effector T cells within the TME. The translational impact is underscored in gemcitabine-resistant PDAC, where hyperactive CTR1 and intensified TANs infiltration create a therapy-refractory condition. Combining CTR1 inhibition with gemcitabine synergistically overcomes this resistance by dual remodelling of the TME.

**Conclusions:**

Our findings shed light on how intracellular copper metabolism-regulating molecules modulate the neutrophil infiltration through the TRAF6/STAT3/CCL2 pathway, and particularly, targeting the copper regulator shows potential in optimising the tumour microenvironment in the treatment of pancreatic cancers.

**Figure:**
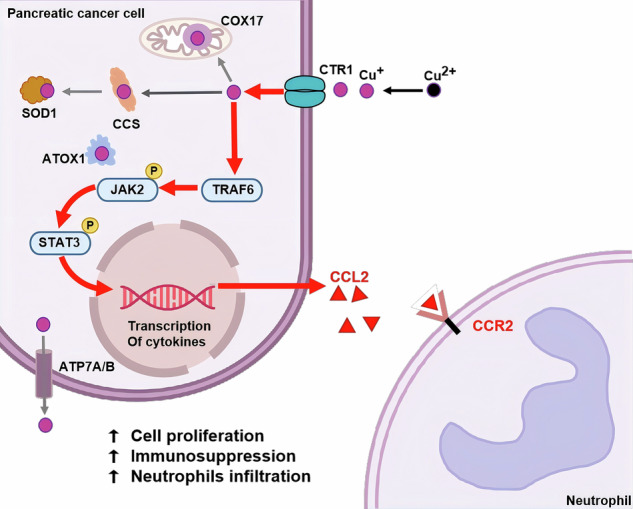
Schematic diagram of the mechanism by which CTR1 regulates neutrophil recruitment through CCL2 transcriptional activation through the TRAF6/JAK/STAT3 Pathway.

## Introduction

Pancreatic ductal adenocarcinoma (PDAC) is an aggressive malignancy representing over 90% of pancreatic cancer cases, yet its 5-year survival rate remains around 10% [1, 2]. Most patients present with advanced or metastatic disease, limiting surgical options. For unresectable cases, gemcitabine chemotherapy is hindered by resistance [3], and the immunosuppressive tumour microenvironment (TME) limits the efficacy of immunotherapy [4].

Recent studies have illuminated the dual role of neutrophils in cancer biology [5]. While they possess inherent antitumour properties, high levels of TANs infiltration correlate with poor prognoses across multiple malignancies [6]. TANs secrete immunosuppressive factors like IL-10, neutrophil extracellular traps(NETs), and TGF-β to inhibit T and NK cells activity [7–9] The interactions between neutrophils and cancer cells, may further release reactive oxygen species (ROS), matrix metalloproteinases, and other cytokines, thereby allow pancreatic cancer cells to evade immune surveillance, enhance tumour invasion and metastatic potential [10]. Evidence suggests that alterations in cytokine and chemokine expression in tumour cells, particularly those from the CXC (Cysteine-X-Cysteine) family, may influence immune cell infiltration [11], yet the precise molecular triggers warrant further exploration.

In addition to immune evasion, PDAC is characterised by aberrant copper metabolism, representing an emerging therapeutic target. While copper is essential for fundamental cellular processes, its dysregulation promotes tumour growth and metastasis across diverse cancers [12–16]. Key molecules in copper metabolism, such as the copper transporter 1 (CTR1), antioxidant protein 1 (ATOX1), and hypoxia-inducible factor 1 alpha (HIF-1α), interact with pathways that regulate tumour initiation and progression [17–19]. Therapeutic use of copper chelators has been demonstrated to inhibit melanoma growth and overcome drug resistance [20]. Additionally, copper’s role in immune function is critical. Deficiencies can impair neutrophil activity and overall immune competency, indicating a complex interplay between copper homoeostasis and the immune landscape in tumours [21, 22]. Recently, a study employing activity-based copper sensing revealed that NRF2-driven cancers exhibit reduced labile copper yet heightened sensitivity to copper chelators, highlighting a cuproplasia-dependent vulnerability that could be exploited in PDAC [23]. Therefore, understanding the multifaceted interactions within the PDAC TME, particularly the roles of neutrophils and copper metabolism, represents a promising avenue for novel therapeutic strategies.

In this study, we establish that CTR1-governed copper metabolism in pancreatic cancer cells directly activates the TRAF6/p-STAT3/CCL2 axis to induce neutrophil infiltration. This cell-autonomous copper signalling initiates tumour microenvironment remodelling by positioning cuproplasia as the primary driver of immune cell infiltration. CTR1 inhibition effectively blocks this copper-to-neutrophil signalling cascade, revealing a targetable axis for pancreatic cancer therapy.

## Methods

### Cell lines and reagents

The cell lines PANC-02, PANC-1, AsPC-1, BxPC-3, SW1990, and PLB-985 were procured from the Chinese Academy of Culture Collection (Shanghai, China), and all cell lines were subjected to STR analysis for authentication. These cells were maintained in Dulbecco’s Modified Eagle Medium (DMEM) enriched with 10% fetal bovine serum (FBS), 2 mM L-glutamine, 100 U/mL penicillin, and 100 μg/mL streptomycin. Culturing was performed at 37 °C within a humidified incubator supplemented with 5% CO_2_.

### Patients and clinical specimens

This research adhered to the guidelines established by the Declaration of Helsinki and its later amendments, or equivalent ethical standards. Approval for the study protocol was granted by the Ethics Committee of Biomedical Research at West China Hospital, Sichuan University. Informed consent was formally waived by the same committee because all samples were de-identified leftover specimens, and the study was retrospective. Human PDAC specimens were obtained from patients who underwent surgical resection at West China Hospital, Sichuan University, during the period from June 1, 2024, to June 30, 2024. Considering the scarcity of surgically resected human pancreatic primary tumours and the need for uniform tissue quality, we rigorously selected 10 high-quality snap-frozen tumour–matched adjacent pairs with complete clinical follow-up data.

### RNA extraction and quantitative reverse transcription-PCR (qRT-PCR)

Total RNA was isolated using TriZol reagent (Invitrogen, California, USA) following the protocol provided by the manufacturer, and the RNA concentration was quantified. For reverse transcription, 2 µg of the extracted total RNA was used in a 20 µL reaction mixture with the commercial reverse transcription kit (Accurate Biology, Changsha, China).

Real-time PCR was performed using the SYBR® Premix Taq kit (Takara Biotechnology, Shiga, Japan) with the following thermal cycling parameters: initial denaturation at 95 °C for 30 s, followed by 39 cycles of denaturation at 95 °C for 5 s and annealing/extension at 60 °C for 30 s. The primer sequences are listed in Supplementary Table 1. Relative gene expression levels were normalised to β-Actin mRNA expression.

### Western blot

Cells and tissues were lysed with RIPA buffer supplemented with 1% protease inhibitors and phosphatase inhibitors. Protein concentrations were measured using the BCA Protein Assay Kit (Vazyme, Nanjing, China). Loading buffer was added to adjust the final volume, and the samples were heated to boiling before proceeding with Western blotting. Proteins (30 μg) were separated by electrophoresis on 10% or 15% SDS-PAGE gels and subsequently transferred to nitrocellulose (NC) membranes. Membranes were blocked with 5% non-fat milk for 1 h and then incubated with primary antibodies overnight at 4 °C. Details of the antibodies and their corresponding catalogue numbers are provided in Supplementary Table 2. After three washes with 0.1% TBST, the membranes were incubated with secondary antibodies (cat. No. L3012 or L3032, Signalway Antibody LLC, USA) for 1 h at room temperature. The blots were visualised using a chemiluminescent substrate (Vazyme, Nanjing, China) and detected with an imaging system (ChemiDoc XRS, Bio-Rad Laboratories, California, USA). The original membranes of the Western blot are available in the Supplementary Material.

### Construction of recombinant plasmids

The shCTR1 sequence was amplified and cloned into vector GV462 (GeneChem, Shanghai, China). The shRNA sequence: *GGAGTACACTTTCATGTGATT*. Lentivirus particles were produced in 293 T cells for stable transfection, and an empty vector was transfected into cells as a control. Then, 2 × 10^7^ TU (Transduction Units) lentivirus supernatant was used to infect 1 × 10^5^ human PDAC cells in a 2 mL culture medium containing 8 μg/mL polybrene (GeneChem, Shanghai, China). After 48 h, 2 μg/mL puromycin (Thermo Fisher Scientific, Waltham, USA) was added for selection.

Depletion of PANC-02 cells was accomplished through transient transfection with the pX459 plasmid containing a small guide RNA (sgRNA) sequence targeting the Cas9 protein. The sgRNA sequence: 5’-*GTGCCCGGCGTCCATGACGT*-3’. The plasmid was constructed by ligating the synthesised oligonucleotide into the linearised pX459 vector using the BbsI restriction enzyme (New England Biolabs, Ipswich, USA). Three distinct sgRNAs were designed based on NGG protospacer adjacent motif (PAM) sequences (https://zlab.bio/guide-design-resources). Forty-eight hours post-transfection, single-cell clones were selected using 1.0 μg/ml puromycin. Screening of single-cell clones was continued in culture. Once the cell cultures reached sufficient confluence, the cells were sequenced and validated by Western blotting.

### Bioinformatics analysis

We downloaded pancreatic cancer data from the TCGA database (https://portal.gdc.cancer.gov/) and performed differential gene expression analysis using the R package DESeq2. After the analysis, we specifically focused on the expression differences of key molecules in the copper metabolism network. We integrated single-cell pancreatic cancer data downloaded from the GEO database (https://www.ncbi.nlm.nih.gov/geo/) (GSE154778, GSE155698) and the CRA database (https://ngdc.cncb.ac.cn/) (CRA001160). The integrated dataset includes 11 normal samples from Peng_2009, 10 primary and 6 metastatic samples from Lin_2020, and 17 primary and 3 normal samples from Steele_2020 [24–26]. The count data were imported into the Seurat single-cell analysis software (v4.1.3; https://github.com/satijalab/Seurat), and quality control was performed on the sequencing libraries to remove outlier cells and genes. Using a standard pipeline with Seurat, cells expressing 200–8000 genes and retaining those with <10% mitochondrial gene expression were selected for downstream analysis, resulting in a gene × cell matrix of 32,738 genes × 85,165 cells for further analysis. Then, the data were normalised using the LogNormalize method, and the top 2000 highly variable genes (HVGs) were selected using the vst method. Principal component analysis (PCA) was performed to identify 30 significant principal components. Clustering was conducted using the shared nearest neighbour (SNN) method with a resolution of 0.5, and the results were visualised using uniform manifold approximation and projection (UMAP). Cell type annotation was performed based on the collected pancreatic marker genes. The UMAP was applied to visualise the single-cell transcriptional profile in 2D space.

To evaluate the functional roles of the identified cell marker genes, we performed enrichment analyses using the R package clusterProfiler (version 4.4.4) under default parameters. The significance of enrichment was determined based on an adjusted *p* value threshold of less than 0.05. This threshold was calculated using the hypergeometric test and corrected via the Benjamini-Hochberg procedure. Gene ontology terms and KEGG pathways meeting this criterion were deemed significantly enriched. The bioinformatics analyses were performed by Chengdu Juyun Biotechnology Co., Ltd. (Chengdu, China).

The Kaplan-Meier Plotter database (https://kmplot.com/analysis) provides a comprehensive evaluation of the impact of 54,675 genes on survival outcomes across 21 different types of cancers. In this study, we utilised the Kaplan–Meier Plotter database to investigate the prognostic significance of the CTR1, TRAF6, JAK2 and STAT3 in pancreatic cancer, with a specific focus on disease-free survival. The expression levels of DMT1, ZNT1, and CD44 in PDAC were assessed using the GEPIA database [27]. The analysis was conducted via the “Expression DIY” module for the PAAD dataset, with a Log2FC cutoff of 1 and a *p* value cutoff of 0.05, using matched TCGA normal and GTEx data as controls. The pan-cancer expression of CTR1 was subsequently investigated.

### Animal experiments

Male C57BL/6 mice (5 weeks old, body weight 16–18 g) were housed under specific-pathogen-free (SPF) conditions with a 12 h light/12 h dark cycle. All animal experiments were conducted in accordance with protocols approved by the Animal Care and Use Committee of Sichuan University. For the orthotopic model, approximately 1 × 10^6^ PANC-02 cells were suspended in a 25 μL PBS and 25 μL Matrigel. C57BL/6 mice were anaesthetised with 1.25% Avertin (2,2,2-tribromoethanol (Aibei Biotechnology, Nanjing, China)), and the fur in the splenic region was removed and disinfected with iodophor. An incision was made 0.5 cm below the left rib cage, and the pancreas was carefully exposed. The tumour cell suspension was then slowly injected into the pancreas, and the incision was closed with sutures. For animal studies, >8 mice per group were initially enrolled for orthotopic surgical implantation. After a 3-week engraftment period, bioluminescence imaging and necropsy were used to exclude animals that failed to develop tumours, displayed ectopic growth, or died from perioperative complications. This stringent filtering yielded five valid mice per group with comparable tumour burden and good health, ensuring internal consistency and statistical reliability of the subsequent analyses. Orthotopic tumour-bearing mice that met the inclusion criteria (uniform BLI signal 5–7 d post-injection, no signs of distress) were randomly allocated to treatment groups. Randomisation was performed with an online random-number generator (random.org); mice were sequentially numbered and assigned to cages corresponding to the randomly generated codes. Investigators were blinded to group allocation during imaging and tumour measurement. Gemcitabine (1.2 mg/animal) [28] was administered via intraperitoneal injection every 3 days for 2 weeks, and 200 μg of anti-Ly6G/IgG [29] was given via intraperitoneal injection every 2 days for the same duration. Twenty-five days after the start of treatment, the mice were euthanized, and the tumours were harvested, weighed, and prepared for further analysis. Throughout the study, the investigators responsible for tumour-measurement and endpoint assessment remained blinded to group allocation; random cage codes were revealed only after the database had been locked.

### Single-cell RNA sequencing

The task was executed by Applied Protein Technology (Shanghai, China), involving the preparation of tumour tissue into a single-cell suspension with a concentration of 500 to 1200 cells per microliter for further processing on the 10x Genomics Chromium™ system. Once the cell count was determined, Gel Bead Emulsions (GEMs) were created for single-cell isolation, followed by reverse transcription using a PCR machine to introduce barcoding. The first-strand cDNA was purified and enriched using magnetic beads and then amplified with subsequent quality control steps to finalise the library preparation. Sequencing was performed on the Illumina NovaSeq 6000 platform using paired-end 150 (PE150) sequencing. Data analysis was conducted using the official 10x Genomics software, Cell Ranger.

### Spatial RNA sequencing

A clinical sample was selected for paraffin embedding, sectioning, and subsequent hematoxylin and eosin (H&E) staining, followed by imaging. Multiple sections were collected for RNA extraction. Captured mRNA was used as a template for reverse transcription, cDNA amplification, and sequencing library preparation. Libraries that passed quality control were sequenced using the Illumina NovaSeq 6000 platform with paired-end (PE) 150 bp reads, targeting approximately 50,000 to 100,000 reads per spot. Finally, the sequencing data were analysed to determine gene expression levels, spatial positions of the detected spots, and the visualisation of the analysis results.

### Inflammatory cytokine array analysis in mice

Serum samples were collected from experimental mice post-euthanasia, and cell culture supernatants were centrifuged to remove pellets. To quantify the levels of inflammatory cytokines, we used a cytokine array kit (QAM-CHE-1, RavBiotech Inc., Guangzhou, China). Samples were thawed on ice and diluted according to the manufacturer’s instructions. The samples were then incubated overnight at 4 °C with microarrays containing capture antibodies specific for the cytokines. After incubation, the membranes were washed several times with wash buffer to remove unbound proteins. Detection antibodies conjugated with fluorescent or enzymatic labels were applied, followed by another round of washing to remove excess reagents. Cy3-streptavidin was then added, and the slides were wrapped in aluminium foil and incubated on a shaker at room temperature for 1 h. Following incubation, the slides were washed again. Signals were detected using a laser scanner (InnoScan 300 Microarray Scanner, Innopsys, France) with Cy3 or green channel excitation (excitation wavelength = 532 nm). Data analysis was performed using the QAM-CHE-1 data analysis software.

### ELISA

The ELISA kit was purchased from Ruixinbio (Quanzhou, China). Cell culture supernatants: Centrifuged at 4000 rpm for 20 min to remove cell debris and polymers. Adherent cells were gently washed with cold PBS, digested with trypsin, and collected after centrifugation at 1000 × *g* for 5 min. For every 1 × 10^6^ cells, resuspend in 150–200 μL PBS and lysed the cells by repeated freezing and thawing. Centrifuge the extract at 1500 × *g* for 10 min and take the supernatant for detection. Add samples to the wells and incubate at room temperature for 1 h. Wash to remove unbound primary antibodies. Add the secondary antibody to the wells. Incubate at room temperature for 1 h. Wash to remove unbound secondary antibodies. Add 100 μL of the substrate solution to the wells and incubate at 37 °C for 15 min. Finally, the stop solution was added, and the absorbance is measured at 450 nm. Compare the absorbance values with those of the standard controls to quantify the amount of target antigen in the samples.

### Flow cytometry

Tissue samples were minced into small pieces and digested with collagenase D containing DNAse I at 37 °C for 1 h. After digestion, the cell suspension was filtered through a 70 μm strainer and washed twice with PBS. Red blood cell lysis buffer was added, and the mixture was incubated at room temperature for 5 min. Cells were resuspended in PBS and stained with flow cytometry antibodies (Supplementary Table 2) at 4 °C for 30 min. Excess antibodies were removed by washing, and the cells were analysed using FlowJo software. Cells were classified based on marker expression, and the proportions of each subpopulation were calculated.

### Neutrophil chemotaxis assay

PLB-985 cells were thawed and cultured following previously described methods. GM-CSF was added to the culture at a concentration of 20 ng/mL. Cell morphology was observed daily, and the differentiation process was documented. The differentiation of neutrophils can be confirmed by detecting cell surface markers such as CD11b and Ly6G using flow cytometry.

The induced PLB-985 cells were resuspended in culture medium and adjusted to a concentration of 2 × 10^6^ cells/mL. Transwell inserts were placed in a 24-well plate. To each well, 600 μL of PDAC cell suspension was added. Then, 100 μL of the resuspended neutrophil cell suspension was added to the upper chamber of each Transwell insert. The plates were incubated at 37 °C and 5% CO_2_ for 12 h. After incubation, the medium from the lower chamber was collected, and the migrated cells were gently resuspended. The number of migrated cells in the lower chamber was counted using a hemocytometer.

### Cleavage under targets & tagmentation (CUT&Tag)

PANC-1 cells were initially treated with formaldehyde solution for fixation to preserve cellular structure and antigen integrity. Subsequently, specific primary antibodies (p-STAT3/IgG/CTCF) and secondary antibody complexes were added to bind with the target proteins. Next, the transposase was activated to cleave DNA fragments in the vicinity of the binding sites. After cleavage, DNA was extracted from the cells and purified to remove impurities and unbound antibodies. Finally, the extracted DNA fragments were analysed by agarose gel electrophoresis to verify the accuracy and specificity of the experimental results.

### Dual-luciferase assay

PANC-1 cells were pre-treated with expression interventions targeting CTR1 and STAT3. Four experimental groups were established: negative control, CTR1 knockdown, STAT3 overexpression, and CTR1 knockdown with STAT3 rescue group. CTR1 knockdown was conducted as previously described. For the STAT3 overexpression plasmid, the CDS sequence of STAT3 was inserted into pVAX1 via seamless cloning.

Cells were transfected with the CCL2 promoter reporter plasmid (pGL3-basic, 200 ng) and the internal control plasmid pRL-TK (20 ng). After 24 h, cells were lysed and luciferase activities were measured using the Dual Luciferase Reporter Assay Kit (Vazyme, China) on a Tecan Spark® microplate reader. Firefly luciferase activity was normalised to Renilla luciferase activity for each sample, and relative promoter activity was calculated compared to the control group.

### Immunohistochemistry (IHC) and immunofluorescence (IF)

The tumour tissue was fixed in 4% paraformaldehyde and embedded in paraffin for routine histopathological examination. IHC was performed to evaluate the expression of CTR1, MPO, TRAF6, p-JAK2, p-STAT3, CCL2, and CD8. All slides were examined under an optical microscope, and quantitative analysis was carried out using ImageJ software. The average optical density (AOD) was measured for statistical analysis, with five randomly selected fields per sample averaged to represent each individual value. For the ten clinical specimens, H-score analysis was conducted using the IHC Profiler plugin. The H-score was calculated based on staining intensity and the percentage of positive cells, yielding a theoretical range of 0 to 300.

Tumour tissue sections were fixed with 4% paraformaldehyde, permeabilized with 0.2% Triton X-100, and blocked with 5% normal goat serum. Samples were incubated with Anti-CTR1, Anti-MPO, Anti-CCR2, Anti-CK19, Anti-CD31 and Anti-α-SMA overnight at 4 °C, followed by incubation with fluorescently labelled secondary antibodies for 1 h at room temperature in the dark. Nuclei were counterstained with DAPI, and sections were mounted with antifade mounting medium. Immunofluorescence was imaged using a confocal microscope. In the cellular immunofluorescence assay, cells were pre-seeded on coverslips. The copper ion probe (MedChemExpress, America) was co-incubated with the cells at 37 °C for 30 min before staining, and the remaining treatment procedures were the same as those for tissue sections. Samples were incubated with Anti-TRAF6, Anti-CTR1, and Anti-CCS at 4 °C overnight, followed by subsequent secondary antibody incubation, DAPI incubation, and imaging.

### ICP-MS assay

Elemental analysis was performed by the Analytical and Testing Center of Sichuan University. All tissue samples were dried at 60 °C for 48 h. Following microwave digestion, the copper content was quantified using inductively coupled plasma mass spectrometry (ICP-MS) (Agilent 7900, California, USA).

### Statistical analysis

This experiment was performed under double-blind conditions. The investigators who administered the treatments were distinct from those who collected and analysed the data. All reagents/devices were labelled with random codes, and the key was held by an independent custodian. Group identities were revealed only after the dataset had been locked. The bars or symbols in the graph represent the mean ± standard error from at least three independent experiments. Data are presented as mean ± standard deviation. The correlation between tissue copper content and CTR1 expression levels was evaluated using Spearman’s rank correlation test. Statistical significance between groups was evaluated using a two-tailed Student’s *t* test or one-way ANOVA, with *p* < 0.05 indicating significance.

## Results

### Aberrant profiles of copper levels and key molecules in PDAC

**Fig. 1:**
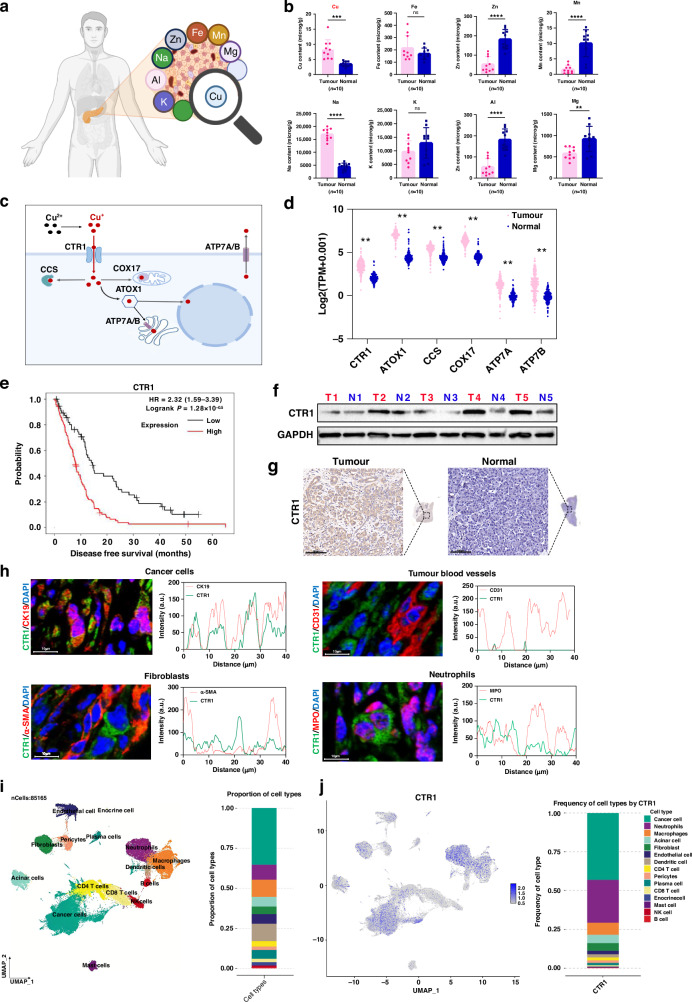
Active copper metabolism and the overexpression of CTR1 have been identified in PDAC. **a** A variety of metallic elements were involved in the development of pancreatic cancer. **b** Multiple metallic elements were measured in ten clinical PDAC samples using ICP-MS. **c** A schematic diagram illustrating the regulation of intracellular copper homoeostasis. **d** Expression of key molecules CTR1, ATOX1, CCS, COX17, ATP7A and ATP7B in pancreatic cancer. **e** The correlation of CTR1 with disease-free survival in pancreatic cancer. **f** CTR1 protein expression was detected in 5 pairs of clinical samples by western blotting. **g** CTR1 expression was assessed by immunohistochemistry in clinical samples. Scale bar = 100 μm. **h** Immunofluorescent staining was performed to detect the distribution of CTR1 expression in tumour cells, vascular endothelial cells, fibroblasts, and neutrophils. Scale bar = 10 μm. **i** Single-cell sequencing of clinical pancreatic cancer samples and cell clustering analysis. **j** Expression levels of CTR1 in different cell types within the pancreatic cancer microenvironment and corresponding statistics. (*p < 0.05, **p < 0.01, ***p < 0.001, Student’s *t* test).

The role of metal elements in the pathogenesis and progression of pancreatic cancer has garnered increasing attention (Fig. 1a). Using inductively coupled plasma mass spectrometry (ICP-MS) to analyse the levels of various metal elements in tumour tissues and paired adjacent normal tissues from 10 PDAC patients. Our results revealed that the concentrations of zinc (Zn), manganese (Mn), and magnesium (Mg) were significantly reduced in PDAC compared to adjacent non-cancerous tissues. Notably, copper (Cu) and sodium (Na) exhibited abnormal accumulation in the tumour microenvironment (Fig. 1b). Given the established critical roles of copper in oxidative stress regulation, angiogenesis, and epigenetic modifications, which are key biological processes associated with cancer [30], we have chosen to focus on abnormal copper metabolism as the entry point for our subsequent research.

Cellular homoeostasis of copper is tightly regulated by specific molecular mechanisms. While several transporters such as Divalent Metal Transporter 1 (DMT1), CD44, and ZNT1 (Zinc Transporter 1) have been implicated in copper uptake [31]. DMT1 exhibits significantly lower copper affinity compared to CTR1 and our results showed that its expression was unaltered in pancreatic cancer (Supplementary Fig. 1A). Beyond its known functions in hyaluronan-dependent adhesion and migration, CD44 also acts as a copper scavenger, concentrating the ion at the membrane surface without facilitating transmembrane transport [32], and its expression showed no statistically significant difference (Supplementary Fig. 1A). ZNT1, primarily a zinc exporter [33], is not considered a canonical copper transporter. Therefore, we focused on CTR1 as the primary and most differentially significant copper transporter for subsequent investigation. Upon entry, copper is sequestered by chaperone proteins including ATOX1, cytochrome c oxidase copper chaperone (COX17), and copper chaperone for superoxide dismutase (CCS), while excess copper is exported via ATPase copper transporting alpha/beta (ATP7A/ATP7B) (Fig. 1c) [13].

To systematically evaluate the copper metabolism network in PDAC, we analysed transcriptomic data from 179 PDAC tissues and 169 adjacent normal tissues from the TCGA database. Critical genes of CTR1, ATOX1, CCS, COX17, ATP7A, and ATP7B, completely exhibited aberrant overexpression in PDAC (Fig. 1d). Given the active copper metabolic network, these PDAC tissues exhibited higher copper accumulation compared to adjacent normal tissues. To identify the most relevant prognostic factor in copper metabolism, we analysed the association of key genes (CTR1, ATOX1, CCS, COX17, ATP7A, ATP7B) with progression-free survival (Supplementary Fig. 1B). This screening revealed CTR1 as the predominant risk factor, displaying the highest hazard ratio (HR = 2.32) for disease progression (Fig. 1e). We found that significantly upregulated CTR1 mRNA and overexpressed protein in 10 primary PDAC tumour tissues compared to the paired adjacent normal tissues, using real-time PCR, immunoblotting, and IHC staining (Fig. 1f, g and Supplementary Fig. 2A–C). And the H-scores of immunohistochemistry results are presented in Table 1. Furthermore, Spearman correlation analysis revealed a strong positive association between CTR1 expression and tissue copper content in these samples (Supplementary Fig. 2D), supporting a direct relationship between CTR1 and copper accumulation in PDAC. To place this finding in a broader context, we performed a pan-cancer analysis of CTR1 expression. The significant upregulation of CTR1 across multiple solid tumours prompted our focus on its role in pancreatic cancer, a malignancy with a distinctive TME where copper metabolism is particularly relevant (Supplementary Fig. 2E).

**Table 1:** The tumour staging and IHC H-score.

Patient No.		TNM staging	H-Score	
			Tumour	Normal
1	T1 N1 M0		156.34	12.32
2	T2 N1 M0		45.37	23.45
3	T2 N0 M0		68.45	42.32
4	T2 N0 M0		94.76	14.32
5	T2 N1 M0		134.54	38.23
6	T2 N0 M0		167.23	26.33
7	T2 N1 M0		59.32	12.30
8	T2 N1 M0		275.32	63.23
9	T2 N0 M0		154.23	51.43
10	T2 N0 M0		137.23	38.32

To examine the cellular localisation of CTR1, immunofluorescent dual-staining was used to identify the localisation of CTR1 expression in specific cell subtypes. We observed that CTR1 exhibited high expression in cancer cells and neutrophils, moderate expression in fibroblasts, and minimal expression in vascular endothelial cells (Fig. 1h). Subsequently, we performed the single-cell sequencing data from 23 published PDAC cases from the GEO database and the CRA database. Consistent with Fig. 1h, CTR1 was predominantly expressed in cancer cells, with neutrophils being the secondary site of expression (Fig. 1i, j).

### Neutrophil depletion attenuates PDAC tumour progression

**Fig. 2:**
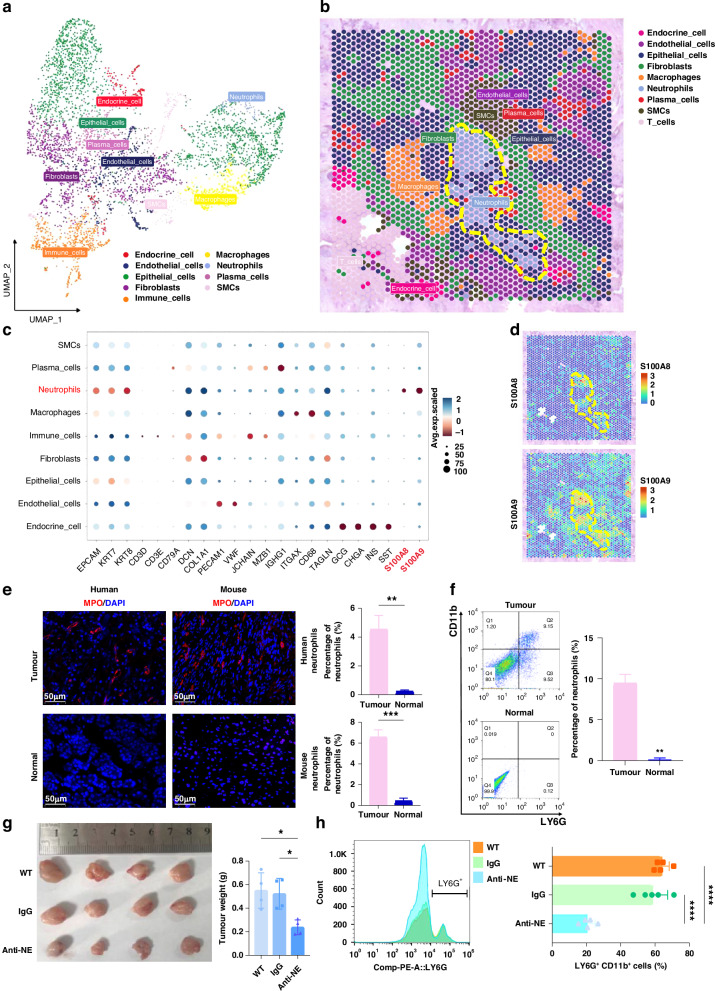
Aberrant neutrophil infiltration is present in pancreatic cancer. **a** The analysis involves multi-sample integration, dimensionality reduction, and clustering using the Seurat software package and the Harmony algorithm. Cell types were identified using classical markers and visualised with a UMAP plot. **b** Spatial transcriptomic analysis of cellular proportions and spatial distribution within the pancreatic cancer microenvironment, revealing region-specific enrichment patterns of immune and stromal cell populations. **c** Dot plot illustrating the two most highly expressed genes across distinct cell types, with dot size representing the proportion of cells expressing the gene and colour intensity indicating the average expression level. **d** Spatial expression patterns of S100A8 and S100A9 in pancreatic cancer tissues, showing co-localised high expression of these myeloid cell markers at the tumour-invasive front. **e** Immunofluorescent staining to detect neutrophil infiltration in human PDAC samples and orthotopic mouse PDAC tissues. Scale bar = 50 μm. **f**, **g** Neutrophil infiltration in mouse tumour tissues was detected using flow cytometry. **g** Mouse pancreatic cancer tumour size after anti-NE treatment. **h** Flow cytometry analysis and quantification of neutrophils in pancreatic cancer tissues from mice treated with IgG and anti-NE.  (*p < 0.05, **p < 0.01, ***p < 0.001, ****p < 0.0001, Student’s *t* test or one-way ANOVA).

To thoroughly characterise the immune microenvironment of PDAC, we generated spatial transcriptomics data from 4833 spots in clinical PDAC tissue, with an average of approximately 88,980 reads and 4235 unique genes detected per spot. Using the Seurat software package and the Harmony algorithm for multi-sample integration, dimensionality reduction, and clustering analysis, we identified 9 clusters and their respective cellular compositions. Subsequently, we utilised classical markers to identify cell types, and the results are shown in Fig. 2a. The labelled cells were mapped to their spatial positions, which revealed the infiltration of multiple immune cell types in PDAC tissues, with significant neutrophil infiltration (Fig. 2b). Then, the dot plot and spatial distribution analysis indicated that two tumour-associated neutrophils (TANs) biomarkers, S100A8 and S100A9 [34, 35], were highly expressed in these infiltrated neutrophils (Fig. 2c, d). Then, neutrophils in human and mouse tissues were marked by myeloperoxidase (MPO) staining, and substantial neutrophil infiltrations were similarly observed in tumour tissues instead of normal pancreatic tissue (Fig. 2e). Thereafter, cell sorting of cells with CD11b^+^/LY6G^+^ biomarkers also revealed substantial neutrophil infiltration in the PANC-02 mouse derived orthotopic tumours while showing minimal neutrophil presence in normal pancreatic tissue (Supplementary Fig. 3). Depletion of neutrophils by anti-NE antibody could effectively inhibit mouse tumour growth (Fig. 2f), indicating that neutrophils in PDAC tend to adopt a tumour-associated neutrophils (TANs) phenotype. Simultaneously, the reduction of neutrophils in the tumour demonstrated the effectiveness of our neutrophil-targeting therapy (Fig. 2g, h). Collectively, these data show that abnormal infiltration of neutrophils occurs in PDAC, and these neutrophils are inclined to promote tumour growth. These advances highlight the potential of neutrophils as therapeutic target in pancreatic cancer.

### CTR1 knockdown inhibits TANs infiltration

**Fig. 3:**
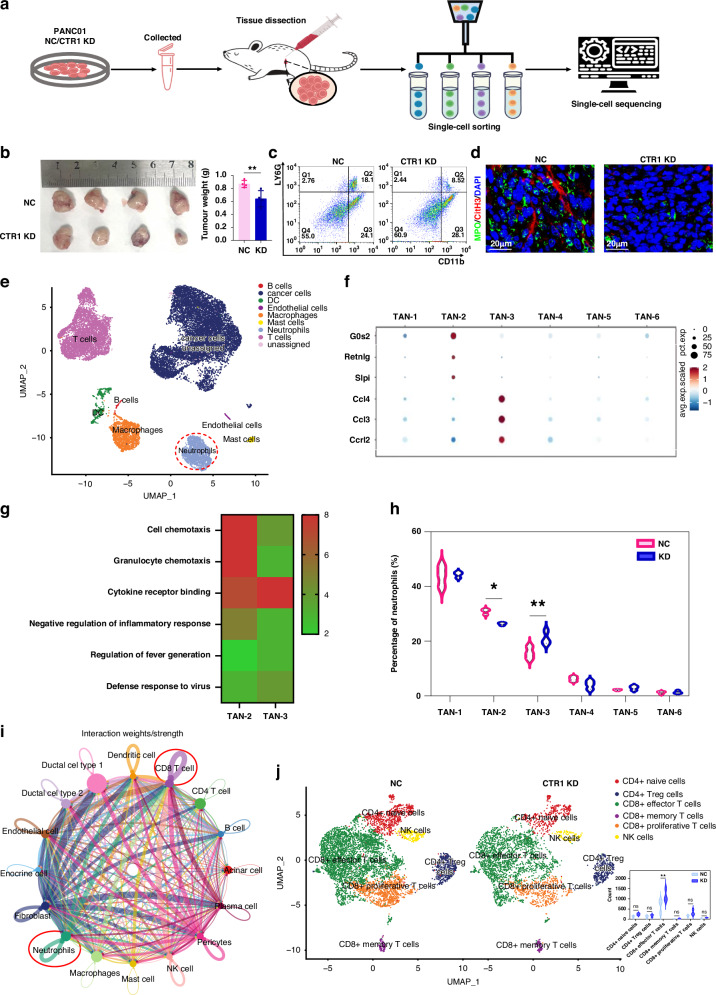
Knockdown of CTR1 in PDAC cells inhibited TANs infiltration in tumour sites. **a** Workflow diagram of PANC-02 cells with CTR1 knockdown for tumour modelling, single cell sorting and analysis. **b** CTR1 knockdown inhibits PDAC growth. **c** Flow cytometry indicates suppressive neutrophil infiltration in dissected pancreatic cancer tissues post CTR1 knockdown. **d** Immunofluorescent staining detects less NETs in pancreatic cancer tissues after CTR1 knockdown. Detection of NETs by simultaneous immunofluorescence staining for MPO and CIT/H3. Scale bar = 20 μm. **e** UMAP visualisation of single-cell RNA sequencing data from orthotopic PDAC tumours, depicting the major cell clusters identified. Neutrophils (highlighted in blue) were identified based on expression of canonical marker genes (S100a8 and S100a9). **f** Dot plot displaying the expression of selected surface marker genes used to characterise the six neutrophil subclusters. The size of the dots represents the percentage of cells within a subcluster expressing the gene, and the colour intensity represents the average expression level. TAN-2 and TAN-3 subpopulations exhibit unique gene expression signatures. **g** All the cells from PDAC tumour tissues annotated as neutrophils were analysed as TANs, including neutrophil clusters 1 to 6. **h** Comparison of pathway activities between the different neutrophil subclusters. The pathways were associated with neutrophil functions. **i** A dot plot was used to illustrate the interactions among various cell types within the mouse pancreatic cancer microenvironment. The size of the dot represents the number of interactions, while the thickness of the connecting lines indicates the strength of the interactions. **j** Single-cell sequencing of T cell subsets in CTR1 KD PANC-02 tumours illustrated by the UMAP representation, and the statistical analysis of the proportions of these subsets. (*p < 0.05, **p < 0.01, Student’s *t* test or one-way ANOVA).

Subsequently, to further investigate the relationship between CTR1 and neutrophils infiltration, we established orthotopic pancreatic tumour models with & without CTR1 knockdown (KD) using PANC-02 cells and analysed the downstream signalling changes (Fig. 3a). CTR1 KD suppressed tumour growth (Fig. 3b) and significantly reduced neutrophil infiltration (from 18.1% to 8.52%) as measured by flow cytometry (Fig. 3c). Immunostaining results (Fig. 3d) showed decrease of neutrophils infiltration and NETs in dissected CTR1 KD tumour tissues, identified NETs is a key functional mediator of CTR1-driven tumour progression, linking copper metabolism to neutrophil-dependent immunosuppression and metastasis. Then, single-cell RNA sorting and sequencing of dissected tumour tissues were performed, single-cell populations clustering was visualised using UMAP for nonlinear dimensionality reduction. After performing dimensionality reduction and clustering analysis on the obtained data, neutrophils were identified for further analysis (Fig. 3e). We next sought to identify the specific neutrophil subpopulations modulated by CTR1 knockdown. Unbiased single-cell RNA sequencing resolved six transcriptionally distinct neutrophil clusters within the tumour microenvironment (Fig. 3f). Two subsets demonstrated particularly relevant expression profiles: TAN-2 was defined by markers associated with neutrophil migration and chemotaxis (G0S2, Retnlg, Slpi), while TAN-3 displayed a signature oriented toward inflammatory and antiviral responses (Ccl3, Ccl4, Ccrl2) (Fig. 3f, g). Strikingly, CTR1 depletion induced a marked shift in the neutrophils landscape, characterised by a pronounced reduction in the pro-infiltrating TAN-2 subset and a concurrent increase in TAN-3 abundance (Fig. 3h). The selective loss of this chemotaxis-competent TAN-2 population provides a compelling cellular mechanism underlying the overall decrease in neutrophil infiltration and NETs previously observed (Fig. 3d). No significant proportional changes were detected in the remaining clusters (TAN-1, -4, -5, -6), which were either unaffected or present in minimal numbers. Together, these findings establish TAN-2 as a key copper-responsive neutrophil subset through which CTR1 signalling promotes neutrophil-driven tumour progression.

In the tumour microenvironment (TME), the interaction between neutrophils and CD8^+^ T cells is intricate and multifaceted, significantly influencing tumour progression and therapeutic efficacy [36]. Recent studies indicate that tumour-associated neutrophils can induce apoptosis in CD8^+^ T cells via TNFα and NO-dependent mechanisms [37]. Similarly, we uncovered pronounced interactions between neutrophils and CD8^+^ T cells within the tumour microenvironment (Fig. 3i). Considering the critical role of CD8^+^ T cells in orchestrating anti-tumour immunity, owing to their capacity to directly eliminate tumour cells and modulate immune responses, it becomes imperative to evaluate their proportion within the T cell population. Thus, we analysed the proportions of various T cell subsets in PANC-02 tumours with and without CTR1 KD, by single-cell sequencing. As illustrated in Fig. 3j, suppression of CTR1 induced statistically increase of CD8^+^ effective T cells by the UMAP representation, indicating the therapeutic synergy potential by modulating CTR1 and intensifying anti-tumour immune effect of CD8^+^ T cells. Further flow cytometry analysis confirmed the increase of CD3^+^ T cells (16.6% vs. 28.4%) and subset CD8^+^ T cells (10.3% vs. 43.9%) within tumour tissues after CTR1 KD (Supplementary Fig. 3). These findings underscore the potential of CTR1 modulation to bolster antitumour immunity in pancreatic cancer by augmenting CD8^+^ T cell activity through its impact on tumour-associated neutrophils.

### CTR1 modulates neutrophil infiltration through TRAF6/STAT3/CCL2 pathway

**Fig. 4:**
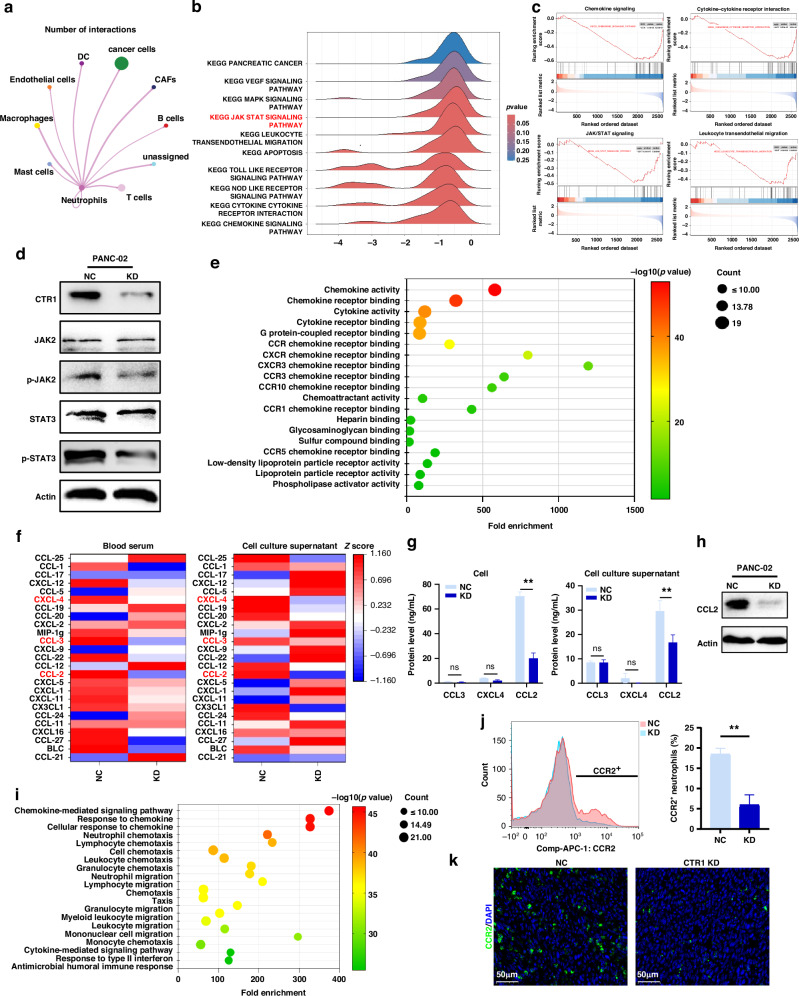
Differential expression of chemokines and receptors connecting cancer cell CTR1 and neutrophils based on PANC-02 cells and orthotopic tumour models. **a** Dot plot showed the correlation between neutrophils and other cells in the pancreatic cancer microenvironment, where the size of the dot represented the number of genes involved in the interaction. **b** KEGG pathway enrichment analyses were conducted to examine differentially expressed genes in orthotopic tumour cells from the NC and KD groups in mice. **c** GSEA enrichment results for the chemokine signalling, cytokine-cytokine receptor interaction, JAK-STAT signalling and leucocyte trans-endothelial migration. **d** Expression of key proteins in the CTR1 mediated JAK/STAT signalling pathway after CTR1 knockdown. **e** GO (Biological Process) pathway enrichment analysis was performed to utilise the screened differentially changed signalling pathways. **f** The heatmap of differentially expressed genes indicated CCL2, CCL3, and CXCL4 were potential key chemokines regulated by CTR1 in PANC-02 cell culture supernatant and blood serum from mice with PANC-02 tumours. **g** ELISA was employed to detect CCL2, CCL3, and CXCL4 secretion in PANC-02 cell lysates and cell culture supernatants. **h** The expression levels of CCL2 in PANC-02 cells were assessed through western blot. **i** Further GO (Molecular Function) analysis was conducted to identify the differential chemotaxis genes in CTR1 KD PANC-02 cells. **j** CCR2 expression of neutrophils from tumour tissue with/without CTR1 knockdown was detected via flow cytometry. **k** CCR2 expression of neutrophils from tumour tissue was visualised using immunofluorescence. Scale bar = 50 μm. (*p < 0.05, **p < 0.01, Student’s *t* test).

Dot plot analysis showed that neutrophils in PDAC were closely related to cancer cells and T cells (Fig. 4a). Then, we used KEGG pathway enrichment analysis to screen differentially expressed genes between NC and CTR1 KD cancer cells. A series of signalling pathways was found to be significantly downregulated (*p* < 0.05) (Fig. 4b). Further GSEA analysis of above pathways focused on the signalling related to neutrophils, and indicated chemokine (NES value −2.734), cytokine-cytokine receptor (NES value −2.671), JAK-STAT (NES value −2.017), and leucocyte trans-endothelial migration (NES value −2.047) were reduced with statistical significance (*p* < 0.01) (Fig. 4c and Supplementary Fig. 4). Considering these results, we performed Western blotting to detect the expression of key proteins in these pathways, and found that p-JAK2 and p-STAT3 were downregulated after CTR1 knockdown in PANC-02 cells (Fig. 4d). Within the tumour microenvironment, STAT3 plays a crucial role, significantly influencing various aspects of tumour biology, including immune cells infiltration [38]. Neutrophil infiltration is usually guided by the chemokine gradients secreted from the tumour cells into the microenvironment. We performed chemokine array assays to evaluate differentially expressed chemokines in the tissues from the mice with PANC-02 tumours after CTR1 knockdown. GO (Biological Process) pathway enrichment analysis revealed that CTR1 knockdown significantly impacted chemokine-related pathways and was strongly associated with neutrophil chemotaxis (Fig. 4e). Subsequently, we evaluated the chemokine genes in both cell supernatants and blood serum, and found that CXCL-4, CCL3, and CCL2 were consistently down-regulated associated to CTR1 KD (Fig. 4f). Furthermore, ELISA and western blot experiments confirmed that the chemokine CCL2 was significantly regulated by CTR1 in both PANC-02 cell lysate and cell culture supernatant (Fig. 4g, h). Molecular Function enrichment analysis highlighted that the effects were related to neutrophil receptors, specifically the CCR and CXCR families (Fig. 4i). In light of our initial findings, we determined the presence of the CCL2/CCR2 ligand-receptor interaction, which plays a significant role in neutrophil chemotaxis [39]. We then used flow cytometry (Fig. 4j) and immunofluorescent staining (Fig. 4k) to examine the expression of the CCR2 on neutrophils collected from the PANC-02 tumour tissue after CTR1 knockdown, which showed a coincident change as expected. These findings point to the fact that hyperactive copper transporter in pancreatic cancer cells would activates the JAK2/STAT3 signalling pathway. Additionally, we also observed that neutrophil infiltration in pancreatic cancer was mediated by the CCL2/CCR2 ligand-receptor interaction.

**Fig. 5:**
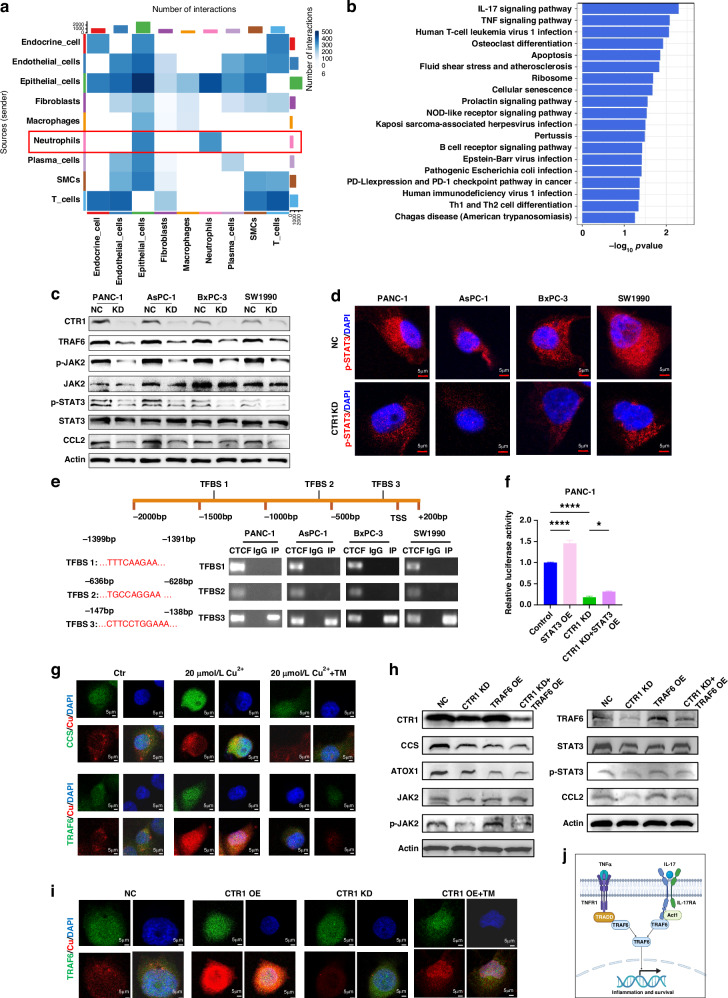
Copper-dependent TRAF6 orchestrates STAT3-mediated CCL2 expression in pancreatic cancer. **a** Correlation analysis of neutrophils with other cells in the human pancreatic cancer microenvironment, with the number of interactions displayed as a heatmap. **b** KEGG pathway enrichment analysis of differentially expressed genes in human tumour cells with varying levels of CTR1 expression (high vs. low). **c** Expression of key proteins in the CTR1 mediated TRAF/JAK/STAT/CCL2 signalling pathway after CTR1 knockdown. **d** Immunofluorescent staining was employed to detect the nuclear translocation of p-STAT3 following CTR1 knockdown. p-STAT3 was visualised with red fluorescence, while nuclei were stained with blue fluorescence. **e** Dual-luciferase reporter assays evaluating CCL2 promoter activity under CTR1 knockdown and STAT3 overexpression conditions. **f** Prediction of STAT3 binding sites on the CCL2 promoter using the JASPAR database, followed by CUT&Tag technology to identify the exact binding sites. **g** Detection of copper binding to CCS and TRAF6 using a copper-specific fluorescent probe following 20 μM CuCl₂ and 60 μM TM treatments. **h** Western blot analysis of CTR1, TRAF6, copper chaperones (ATOX1, CCS), and JAK2/STAT3/CCL2 pathway proteins under TRAF6 overexpression and CTR1 knockdown. **i** Assessment of TRAF6-copper association following CTR1 regulation and TM treatment. **j** Schematic illustration of TRAF6 as the key downstream node integrating TNF and IL-17 signalling to promote STAT3 transcriptional activity. (*p < 0.05, **p < 0.01, Student’s *t* test).

Elevated expression of CTR1 in pancreatic cancer cells would lead to increased secretion of chemokines, which further induces neutrophil recruitment to the tumour site, involving the CCL2/CCR2 interaction. To validate the existence of potential influence of CTR1 in human PDAC, we conducted the neutrophils transwell migration evaluation in four kinds of human pancreatic cancer cells, PANC-1, AsPC-1, BxPC-3, and SW1990. Supplementary Fig. 5A, B indicated suppression of neutrophil migration after CTR1 knockdown. Correlation heatmap of clinical pancreatic cancer tissue showed that neutrophils were regulated by the epithelial cancer cells (Fig. 5a). KEGG pathway enrichment analysis of these samples further identified IL-17 and TNF signalling as the most significantly altered pathways associated with CTR1 dysregulation (Fig. 5b). Given that both IL-17 and TNF signalling converge on TRAF6 [40, 41], and previously identified JAK-STAT cascade (Fig. 4b), we focused on TRAF6 as a pivotal convergent node. We next examined key proteins in this network. Western blot analysis confirmed that CTR1 knockdown downregulated TRAF6, p-JAK2, p-STAT3, and CCL2 across all PDAC cell lines tested (Fig. 5c). Similarly, at the protein level, we detected a decrease of secreted CCL2 in the supernatant of all these cells (Supplementary Fig. 6A, B). Immunofluorescent staining confirmed that the transcriptionally active p-STAT3 in the cytoplasm and nucleus was reduced following CTR1 KD (Fig. 5d). As anticipated, the activation of key proteins within the TRAF6/JAK2/STAT3/CCL2 signalling cascade was significantly inhibited, concomitant with a marked reduction in CCL2 protein expression. Based on the JASPAR database prediction of three potential STAT3 binding sites within the CCL2 promoter region, Cut&Tag assays confirmed specific STAT3 binding at the −147 to −138 bp site upstream of the transcription start site (Fig. 5e). Functionally, CTR1 knockdown reduced CCL2 promoter activity in dual-luciferase assays, while STAT3 overexpression enhanced promoter activity and effectively reversed the inhibitory effect of CTR1 knockdown (Fig. 5f). Notably, clinical survival analysis data further revealed that high expression levels of TRAF6, STAT3, and CCL2 were significantly associated with a shorter disease-free survival period in pancreatic cancer patients (Supplementary Fig. 6C). These findings align with observed suppression of the TRAF6/JAK2/STAT3 signalling cascade and corresponding decreases in both CCL2 promoter activity and protein expression following CTR1 depletion.

We focused our investigation on this critical signalling node. To elucidate the mechanistic basis of copper-mediated tumour progression, we sought to determine whether TRAF6 activation depends specifically on CTR1 protein function or broadly on intracellular copper availability. Using a copper-specific fluorescent probe with the copper chaperone CCS as a reference, we found that 20 μM CuCl₂ treatment enhanced CCS expression and increased copper fluorescence, showing clear colocalization. This effect was reversed by copper chelator tetrathiomolybdate (TM), confirming copper specificity. Notably, TRAF6 displayed a similar response pattern to CCS following copper stimulation (Fig. 5g), indicating that intracellular copper levels play an important role in TRAF6 activation, potentially through CTR1-mediated copper uptake. Then, we performed simultaneous knockdown and overexpression of TRAF6 to delineate its relationship with CTR1-mediated copper signalling. Our results demonstrated that CTR1 knockdown reduced the expression of copper chaperones CCS and ATOX1, while TRAF6 overexpression similarly suppressed their levels. The combined intervention led to the most pronounced reduction (Fig. 5h). Notably, TRAF6 overexpression did not significantly alter CTR1 expression, suggesting that CTR1 likely functions upstream of TRAF6 in the regulatory hierarchy. Furthermore, TRAF6 overexpression activated the downstream JAK2/STAT3/CCL2 axis, whereas CTR1 knockdown effectively reversed this activation (Fig. 5h). To investigate the copper-dependent regulation of TRAF6, we modulated CTR1 expression in PANC-1 cells. Overexpression of CTR1 resulted in elevated intracellular copper levels and augmented TRAF6 activation, as indicated by their pronounced colocalization. Conversely, CTR1 knockdown diminished both intracellular copper content and TRAF6 activation. Critically, copper chelation with TM abrogated the enhancement of TRAF6 activation in CTR1-overexpressing cells (Fig. 5i). Collectively, these findings establish TRAF6 as the key regulatory node that transduces CTR1-mediated copper signalling to drive STAT3-transcriptional activation in pancreatic cancer (Fig. 5j).

### Targeting CTR1 synergises with gemcitabine by enhancing T cell immunity

**Fig. 6:**
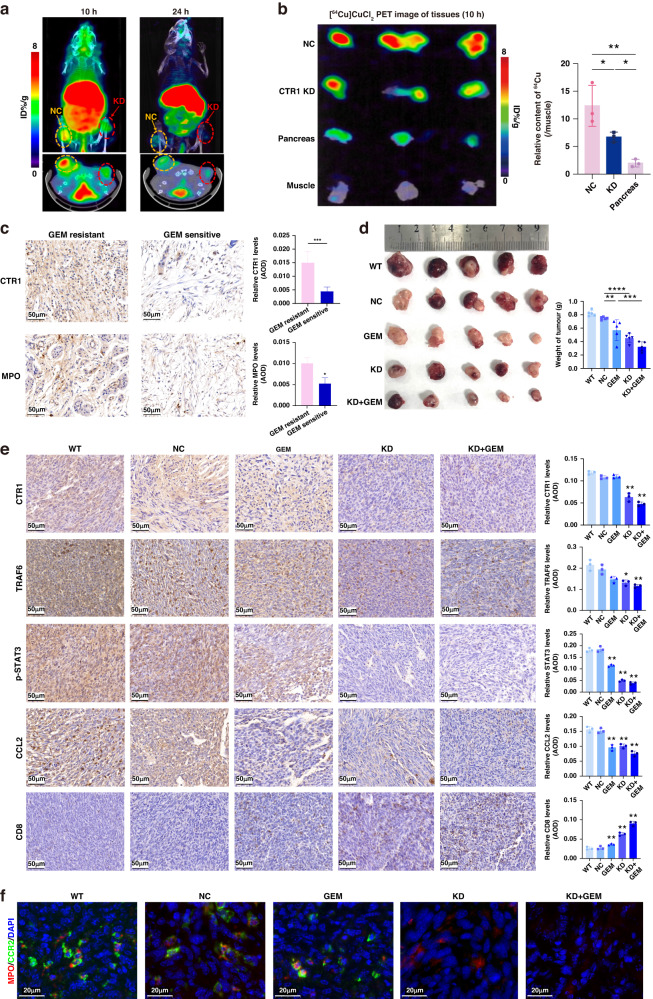
Therapeutic synergy of CTR1 blockage improves gemcitabine treatment in PDAC. **a** Representative microPET/CT imaging of [^64^Cu]CuCl₂ demonstrates that the CTR1 knockdown effectively suppresses the copper uptake and tumour progression in vivo. Yellow arrows indicate the NC PANC-02 tumour while the red arrows indicate the CTR1-KD tumour. **b** The uptake of [^64^Cu]Cu^2+^ ions in dissected tissues from mice bearing orthotopic PANC-02 tumours were evaluated (*n* = 3). **c** IHC analysis indicates elevated expression of CTR1 and MPO in gemcitabine-resistant clinical specimens compared to gemcitabine sensitive specimens (*n* = 5). **d** Harvested tumours and tumour weights were recorded at the end of the treatment. **e** Immunohistochemical staining of CTR1/TRAF6/STAT3/CCL2 and CD8 in tumour tissues from above groups. Scale bar = 50 μm. **f** Immunofluorescent staining of neutrophils and CCR2 expression in tumour tissues from above groups. Scale bar = 20 μm. (*p < 0.05, **p < 0.01, ***p < 0.001, ****p < 0.0001, one-way ANOVA).

Using subcutaneous and orthotopic pancreatic tumour models with CTR1-knockdown PANC-02 cells, [⁶⁴Cu]CuCl₂ PET/CT imaging showed reduced copper uptake in CTR1-KD tumours. At 10 h post-injection, the subcutaneous KD tumour was smaller and had lower copper uptake than the NC tumour (Fig. 6a). Twenty-four hours later, as copper was metabolised, the imaging signal weakens. Orthotopic cancer PET/CT imaging revealed that NC tumours exhibited higher copper uptake, with the NC group showing approximately 1.7 times the uptake of the KD group. Both groups exceeded the uptake level of normal pancreas tissue. Meanwhile, muscle tissue displayed minimal signal (Fig. 6b). CTR1 knockdown thus impairs tumour copper uptake, confirming its role in copper transport and supporting CTR1-targeted therapy development. Gemcitabine continues to be the cornerstone of first-line therapy for pancreatic cancer. Nonetheless, the prevalence of gemcitabine resistance significantly impedes long-term survival outcomes. To address these issues and enhance therapeutic effects, combination therapies involving gemcitabine and other agents, such as targeted therapies or immunotherapies, have been extensively explored [42]. To explore the association between the tumour microenvironment and copper metabolism in gemcitabine-resistant settings, we analysed MPO and CTR1 expression through immunohistochemical staining. Five paired clinical specimens of gemcitabine-resistant and -sensitive pancreatic cancers were examined. Our analysis suggested that gemcitabine-resistant tumours revealed enhanced neutrophil infiltration and elevated CTR1 expression (Fig. 6c and Supplementary Fig. 7). These results suggest a potential relationship between neutrophil infiltration and copper transporter activity in chemotherapy resistance. Interestingly, knockdown of CTR1 showed a marked tumour inhibiting capability to combined treatment with gemcitabine (Fig. 6d) (*n* = 5, *p* < 0.001). Following immunohistochemistry (IHC) results revealed that the expression of STAT3 and CCL2 was markedly reduced in the combination group compared to single-agent treatments, while there was no significant difference in the expression of CTR1 and TRAF6 in the gemcitabine (GEM) group, which suggests that CTR1 and gemcitabine may act on STAT3 through different pathways (Fig. 6e). Additionally, immunofluorescence (IF) results showed a significant reduction in neutrophil infiltration and decreased expression of the neutrophil surface chemokine receptor CCR2 in tumour tissues after combination therapy (Fig. 6f). These results delineate that modulating the CTR1/TRAF6/STAT3/CCL2 signalling cascade can enhance immune responses within the pancreatic cancer microenvironment, contributing to suppressing TANs infiltration alongside a notable increase in CD8^+^ T cells. These observations point towards a promising new direction for enhancing immunotherapy in pancreatic cancer by targeting CTR1 and potentially altering the balance between neutrophils and CD8^+^ T cells.

## Discussion

The connection between copper and cancer has been noted for decades [13]. Dysregulated copper metabolism has a role in tumorigenesis, including hyperplasia, metaplasia and neoplasia [43, 44]. In agreement with findings for hepatic and prostate cancer, elevated copper levels were observed in pancreatic tumour tissues [45, 46]. Our earlier work illustrated that abundant copper within tumours provides a foundation for the excessive growth of pancreatic tumour cells through the AKT/mTOR pathway [47]. However, the subsequent stress of cuproplasia to the tumour microenvironments remains unclear. In this study, we found that overexpressed copper transporter-1 (CTR1), which is the gatekeeper gene of intracellular copper metabolism, was enriched in both cancer cells and neutrophils in PDAC tissues. These findings provide insight into the relationship between cuproplasia and the tumoural immune microenvironment. Then, we found that copper depletion by knocking down CTR1 in pancreatic cancer would interfere with the intracellular signalling axis of CTR1/TRAF6/p-STAT3/CCL2 to suppress tumour-associated neutrophil infiltration, with significant suppression of subtypes associated with neutrophil chemotaxis in pancreatic cancer progression. The observed alterations in the immune landscape following CTR1 targeting point to its potential to create a context that may improve gemcitabine efficacy, a promising approach that warrants further investigation.

The tumour microenvironment (TME) in pancreatic cancer is renowned for its complexity and immunosuppressive nature, and significantly contributes to tumour progression, poor prognosis, and resistance to therapy [48]. The development of the TME is primarily driven by interactions between tumour cells and immune cells. TME secretes immunosuppressive molecules, including TGF-β and IL-10, which inhibit CD8^+^ T cell growth and activation, reducing their cytotoxic effect on tumour cells [49, 50]. Additionally, the interaction of cancer cells and immune cells is mediated by a variety of chemokines to recruit myeloid-derived suppressor cells (MDSCs), exacerbating the immunosuppressive microenvironment [51]. Neutrophil is one of the most important constituents in TME. Interestingly, previous studies have shown that neutrophils have both pro-tumourigenic and anti-tumourigenic effects in different cancers [5, 52, 53]. To investigate this duality, we administered anti-NE antibodies to deplete peripheral blood neutrophils. TANs in PDAC tend to adopt a phenotype associated with tumour growth, while blocking the neutrophil activity would improve the therapeutic outcomes, which resonates with recent research advancements [54, 55]. The homeostasis of trace elements is important for maintaining the immune microenvironment. Hyperactive copper metabolism is involved in tumour growth and metastasis across multiple cancer types, and copper depletion has shown promise as a therapeutic strategy [43]. Several studies revealed that copper has emerged as a critical factor to maintain the immune microenvironment and boosts the effectiveness of immunotherapy in neuroblastoma and breast cancers, but its mechanisms of action require further investigation [56, 57]. Our studies highlight the role of CTR1, a copper transport protein, in the regulation of tumour-associated neutrophil (TAN) infiltration in pancreatic cancer. Interestingly, we observed that copper depletion by blocking CTR1 in pancreatic cancer suppresses neutrophil infiltration, with significant suppression of subtypes associated with neutrophil chemotaxis. Given the challenges in manipulating neutrophils in vitro, we focused on the high expression of CTR1 in PDAC cells to investigate the mechanisms of copper metabolism in PDAC progression. In this study, the inherent difficulties with maintaining neutrophils in vitro and manipulating their genetic content were circumvented by concentrating on PDAC cell lines, which allowed for a more feasible experimental approach to explore the role of CTR1 in copper homeostasis and its implications for the disease’s advancement.

Previous studies have shown that the dysfunction of the copper metabolism network mediated by CTR1 would affect JNK, p53, MAPK, and VEGF signalling pathways involved in cancer cells themselves [58]. However, we found that the CTR1 network can regulate TANs infiltration and TME via the TRAF6/JAK/STAT3/CCL2/CCR2 pathway (Graphical Abstract).

The pancreatic cancer TME also plays a significant role in chemotherapy resistance, a phenomenon termed environment-mediated resistance [59, 60]. The dense extracellular matrix (ECM) in the TME can compress the blood vessels in the tumour tissue, affecting the entry and distribution of chemotherapy drugs [61]. Additionally, targeting ESE3/EHF with Nifurtimox inhibits the infiltration of CXCR2^+^ neutrophils and overcomes resistance to chemotherapy and immunotherapy in pancreatic cancer [62]. Gemcitabine, the primary chemotherapy agent for pancreatic cancer, has garnered attention for its potential to enhance immunotherapy [63]. While gemcitabine alone exhibits limited efficacy, it can augment T-cell cytotoxicity when combined with immunotherapeutic agents like PD-L1xCD3 bispecific T-cell engagers (BiTE) [64]. However, subsequent studies reveal that gemcitabine increases neutrophil infiltration and may promote pancreatic cancer metastasis via the Gas6/AXL signalling pathway, posing a challenge to its immunotherapeutic applications [65]. Since tumour cells secrete relatively low levels of IL-17 and TNF-α, we focused on the downstream pathways of these signals. Typically, IL-17 binds to its receptor IL-17R, leading to the formation and activation of the receptor complex. The activated IL-17R complex recruits TRAF6 to activate multiple downstream signalling pathways, including NF-κB, MAPK, and JNK (c-Jun N-terminal Kinase) [66, 67]. TRAF6 is a key member of the TRAF protein family, which interacts with the TNF receptor superfamily to mediate signal transduction [68]. The overexpression of TRAF6 promotes the degradation of IRF3 through ubiquitination, which reduces the cancer cells’ sensitivity to 5-FU [69], and acts as an upstream regulator of PD-L1 to contribute to tumour immune escape [70]. One of the most sensitive downstream effectors of TRAF6 is the JAK/STAT pathway, which plays a pro-tumour role and strongly suppresses anti-tumour immune responses [71–73], [38]. TRAF6 can inhibit the pathway by mediating STAT3 ubiquitination or activate tumour-associated macrophages through TRAF6/IL-6/STAT3 axis [74]. These findings prompted us to investigate the immunosuppressive and high-copper environment of pancreatic cancer from a new perspective. In our study, we observed that TRAF6 exhibits a promotive effect on the JAK/STAT3 pathway in pancreatic cancer. We speculate that this functional divergence may stem from differences in the nature of upstream signals received by TRAF6 across distinct cellular contexts. Unlike traditional immune signalling stimuli, our findings suggest that CTR1-mediated copper influx may act as a non-canonical upstream signal, potentially inducing specific conformational changes in TRAF6 or recruiting unique protein interaction networks, thereby switching its regulatory effect on the JAK/STAT3 signalling axis from suppression to promotion. This discovery offers a new perspective for understanding the functional regulation of TRAF6 within the TME, while the precise molecular mechanism by which copper modulates TRAF6 activity warrants further investigation. Subsequently, our findings suggest that CTR1 knockdown reduces TANs infiltration while increasing the activation of cytotoxic CD8^+^ T cells, partially mitigating gemcitabine’s neutrophil-related side effects. Reducing copper levels by suppressing CTR1 can block neutrophil infiltration, boost CD8^+^ T cells, and improve the immune environment, suggesting a potential strategy to overcome some limitations of gemcitabine in PDAC treatment. While our findings highlight CTR1 as a promising therapeutic target, several key questions remain open for future investigation. Translational development will require the design of specific CTR1 inhibitors or delivery systems suitable for clinical use. Additionally, our single-cell data indicate that CTR1 is also highly expressed in neutrophils, suggesting that cell-intrinsic copper metabolism may influence neutrophil function within the tumour microenvironment, a compelling aspect that remains to be elucidated. Further studies exploring CTR1 roles across diverse cellular compartments, including myeloid subsets (neutrophils, macrophages, MDSCs), lymphoid populations (T cells, NK cells), as well as cancer-associated fibroblasts, will markedly broaden our understanding of copper-mediated immunoregulation in PDAC.

In conclusion, our findings provide insight into the regulation of tumour-associated neutrophils (TANs) in the PDAC microenvironment. We demonstrate that the copper transporter CTR1 promotes CCL2 secretion via activation of the TRAF6/JAK/STAT3 signalling pathway, suggesting a potential link between copper metabolism and the pathological accumulation of TANs. The proposed crosstalk between copper homeostasis and immune modulation not only enhances our understanding of PDAC pathogenesis but may also offer a conceptual basis for future therapeutic strategies targeting metallo-immunological pathways.

## Supplementary information

Supplementary figure and figure legends

Supplementary table 1

Supplementary table 2

## Data Availability

The datasets used and/or analysed during the current study are available from the corresponding author on reasonable request.
