# Supplementary material for: Blockage of cuproplasia inhibits pancreatic tumour-associated neutrophils infiltration through TRAF6/STAT3/CCL2 pathway
Source: Br J Cancer. 2026 Apr 14;135(1):17–32. doi: 10.1038/s41416-026-03371-8 (PMC13269755; doi:10.1038/s41416-026-03371-8)
Supplement: Supplementary file 1 — Supplementary figure and figure legends [file 41416_2026_3371_MOESM1_ESM.docx]

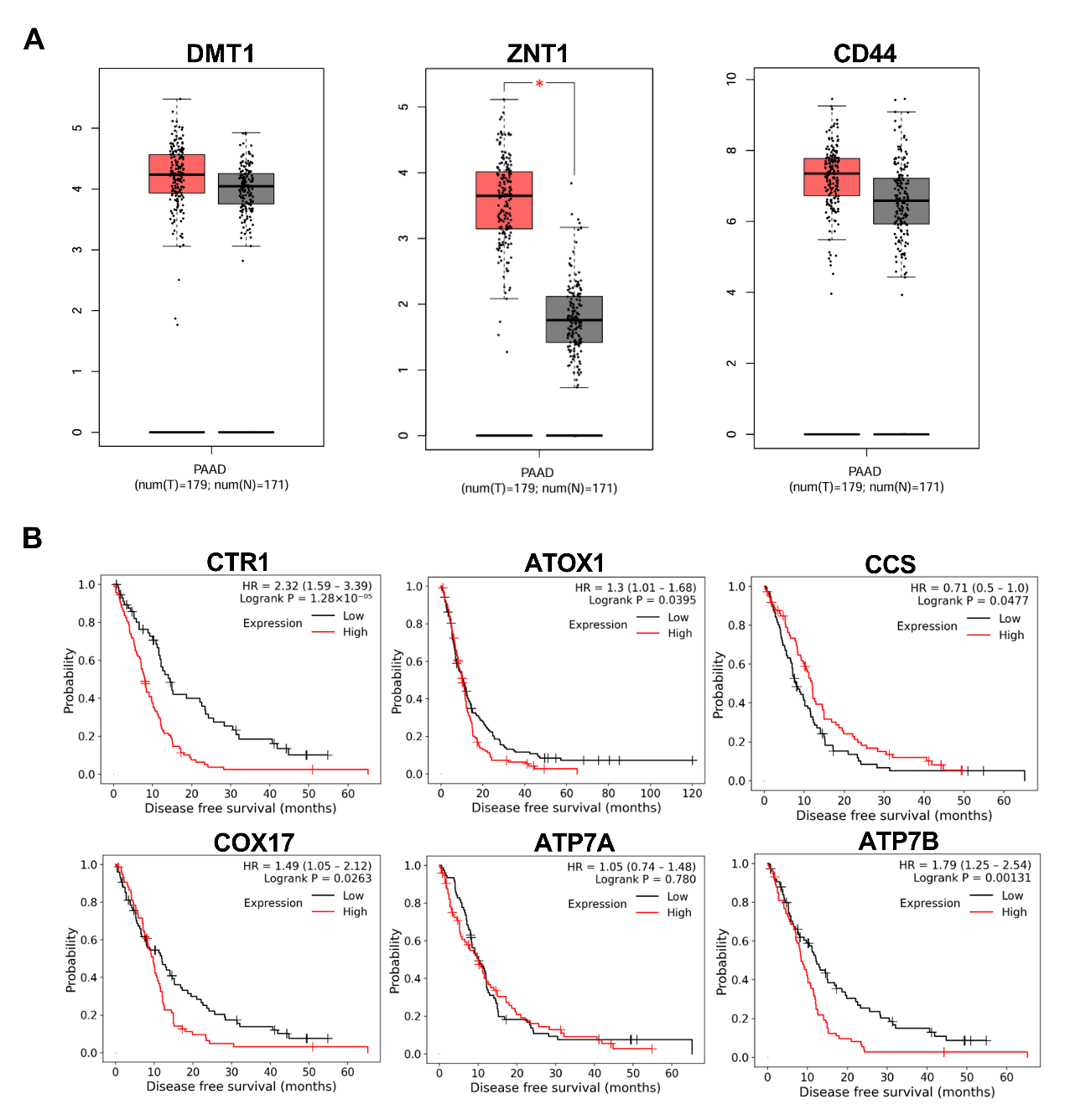


**Supplementary Figure 1.** CTR1 is a prognostic risk factor in pancreatic cancer. (A) Expression differences of DMT1, ZNT1, and CD44 in 179 pancreatic cancer samples were analyzed using the GEPIA database (http://gepia.cancer-pku.cn/). (B)Utilizing the survival analysis website (<https://server2.kmplot>.com/) to investigate the correlation between key genes of copper metabolism and survival in clinical pancreatic cancer. (**P*<0.05, Student’s t-test)


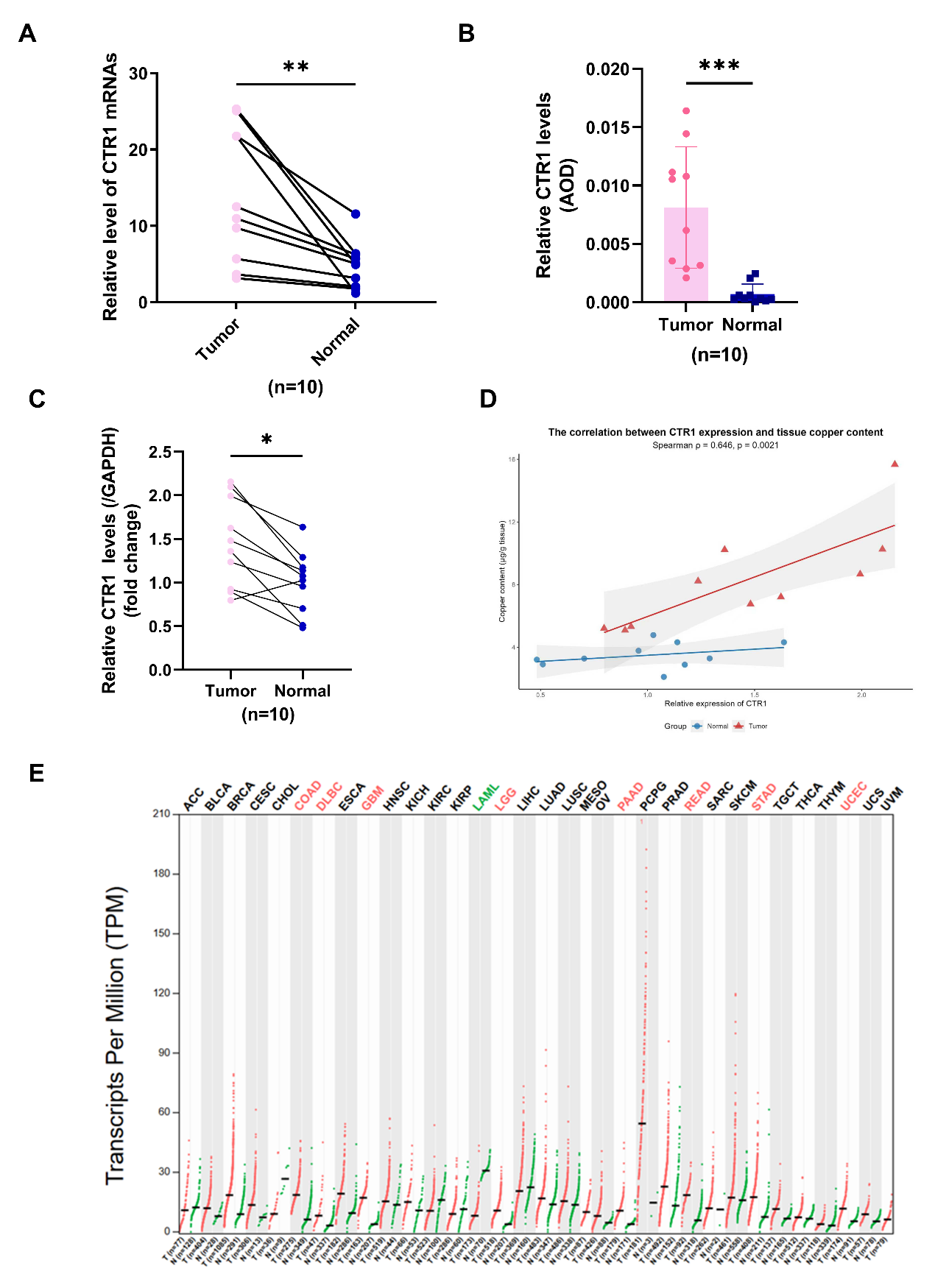


**Supplementary Figure 2.** CTR1 was upregulated in PDAC clinical samples. (A) mRNA levels of CTR1 were measured in 10 pairs of clinical samples. (B) CTR1 protein expression was detected in 10 pairs of clinical samples by western blotting. (C) CTR1 expression was assessed by immunohistochemistry in 10 pairs of clinical samples. (D) Spearman correlation analysis between CTR1 expression levels and copper content in 10 paired PDAC samples. (E) Pan-cancer analysis of CTR1 differential expression using the GEPIA database. (**P<0.01, ***P<0.001, student’s t-test)


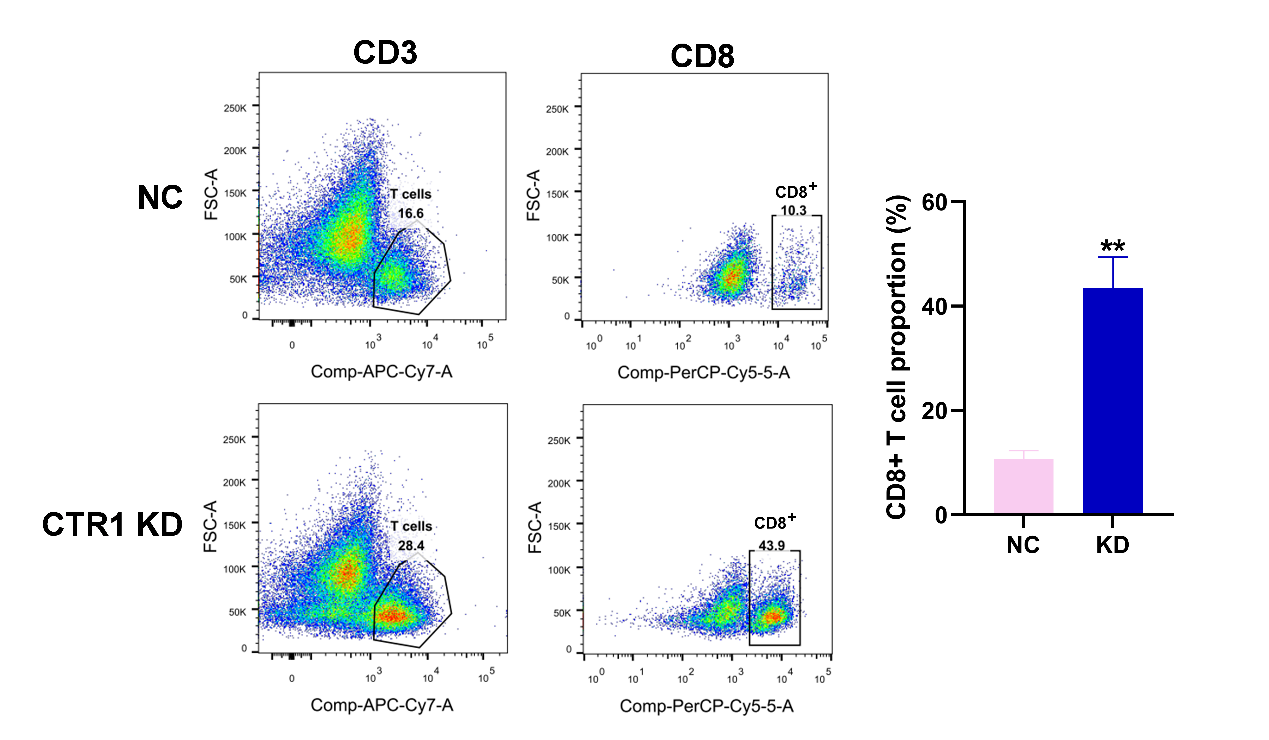


**Supplementary Figure 3.** Flow cytometry was used to assess the proportions of T cells and CD8^+^ T cells following CTR1 knockdown, with CD3 utilized as a marker for T cells and CD8 for cytotoxic T cells. (***P*<0.01, student’s t-test)


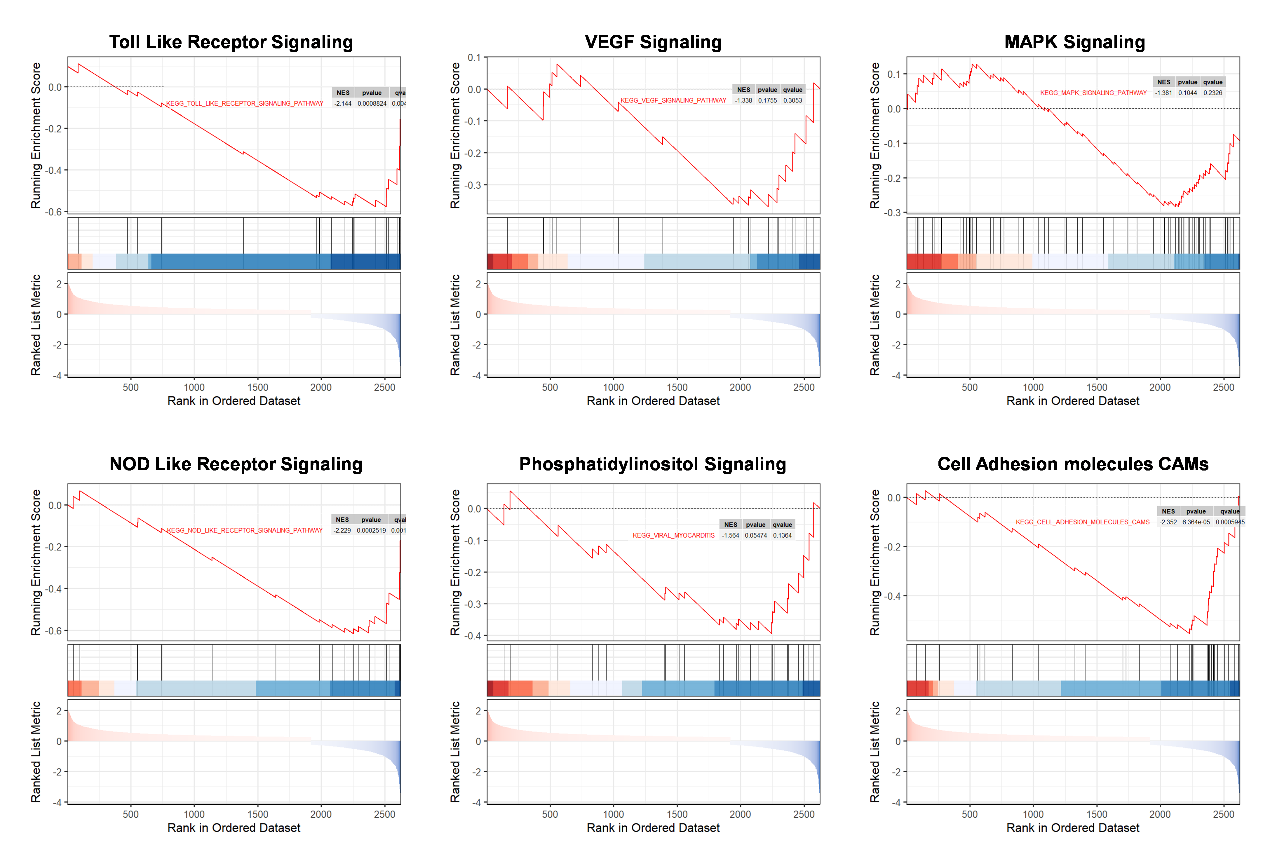


**Supplementary Figure 4.** GSEA analysis of above pathways focused on the signaling related to neutrophils, including Toll-like receptor signaling (NES value -2.144), VEGF signaling (NES value -1.338), MAPK signaling (NES value -1.381), NOD-like receptor signaling (NES value -2.229), Phosphatidylinositol signaling (NES value -1.544), and Cell adhesion molecules (NES value -2.362).


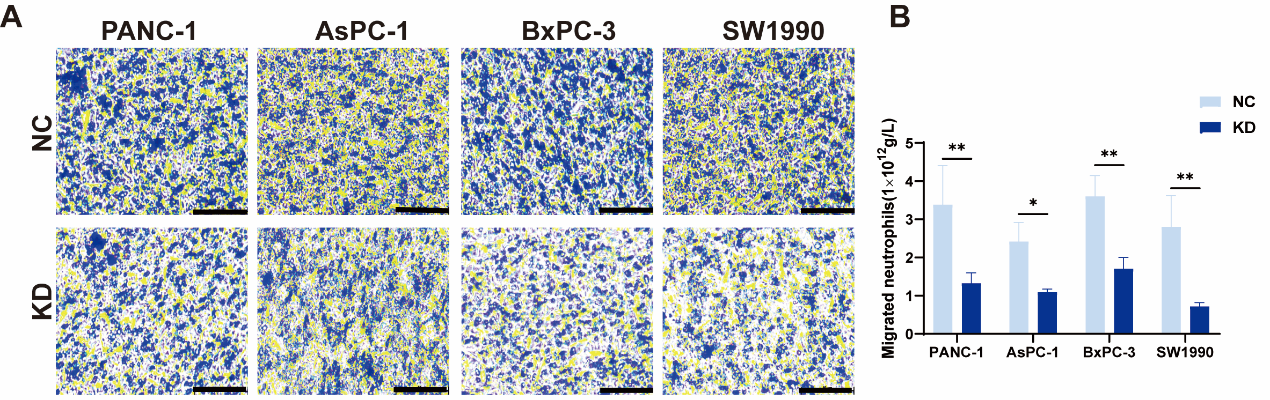


**Supplementary Figure 5.** CTR1 regulates neutrophil migration. (A) Hematoxylin staining (blue) of neutrophils arrested in the transwell membrane following CTR1 knockdown in human pancreatic cancer cell lines (PANC-1, ASPC-1, BXPC-1, and SW1990). (B) Quantification of neutrophils migrated to the lower chamber in the transwell assay.


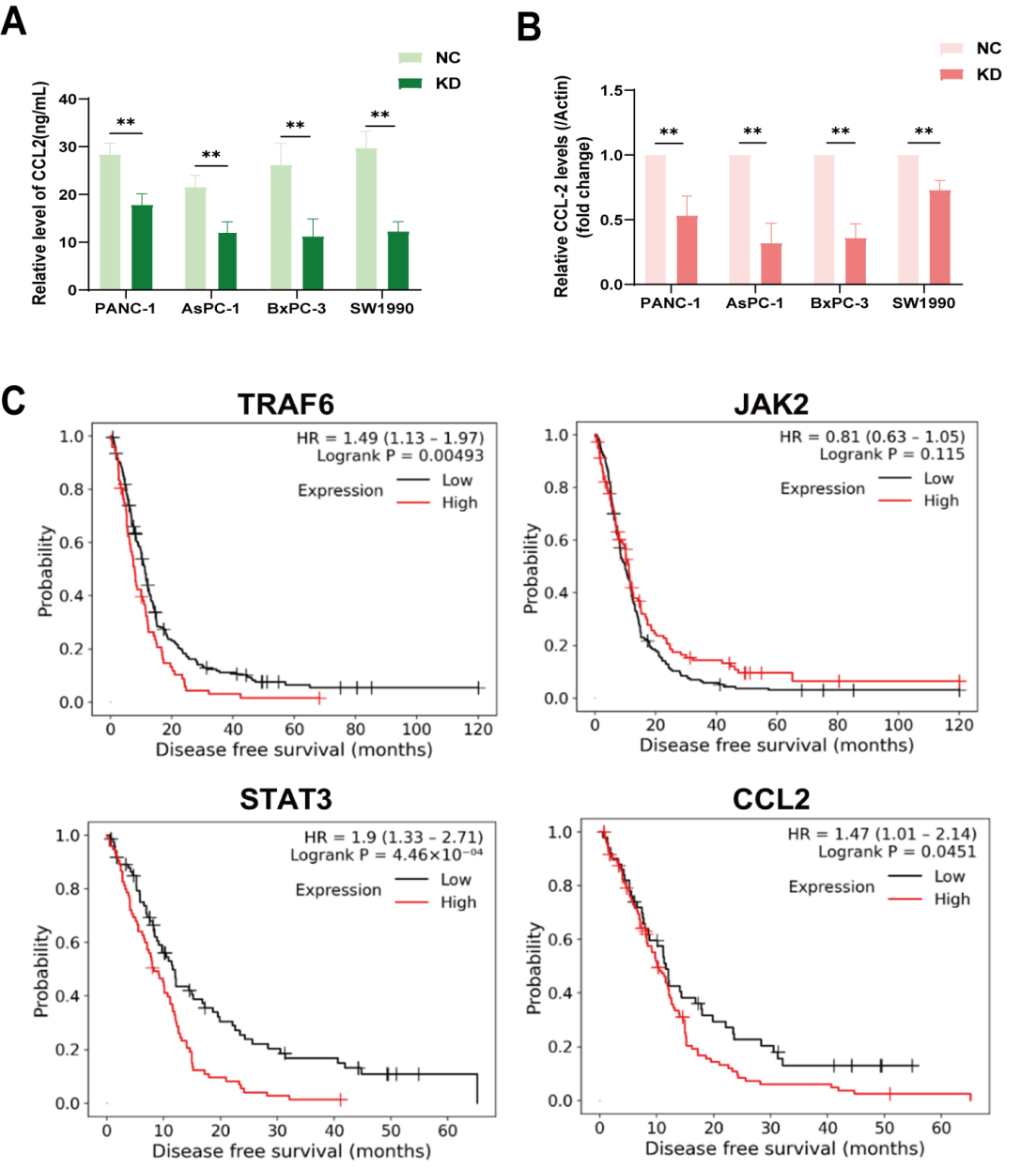


**Supplementary Figure 6.** Prognostic association of the TRAF6/JAK2/STAT3 pathway. CCL2 expression at protein levels(A) and mRNA(B) was detected after CTR1 knockdown using qPCR and ELISA. (C)Utilizing the survival analysis website (<https://server2.kmplot>.com/) to investigate the correlation between key genes in the TRAF6/JAK2/STAT3 pathway and survival in clinical pancreatic cancer.

**
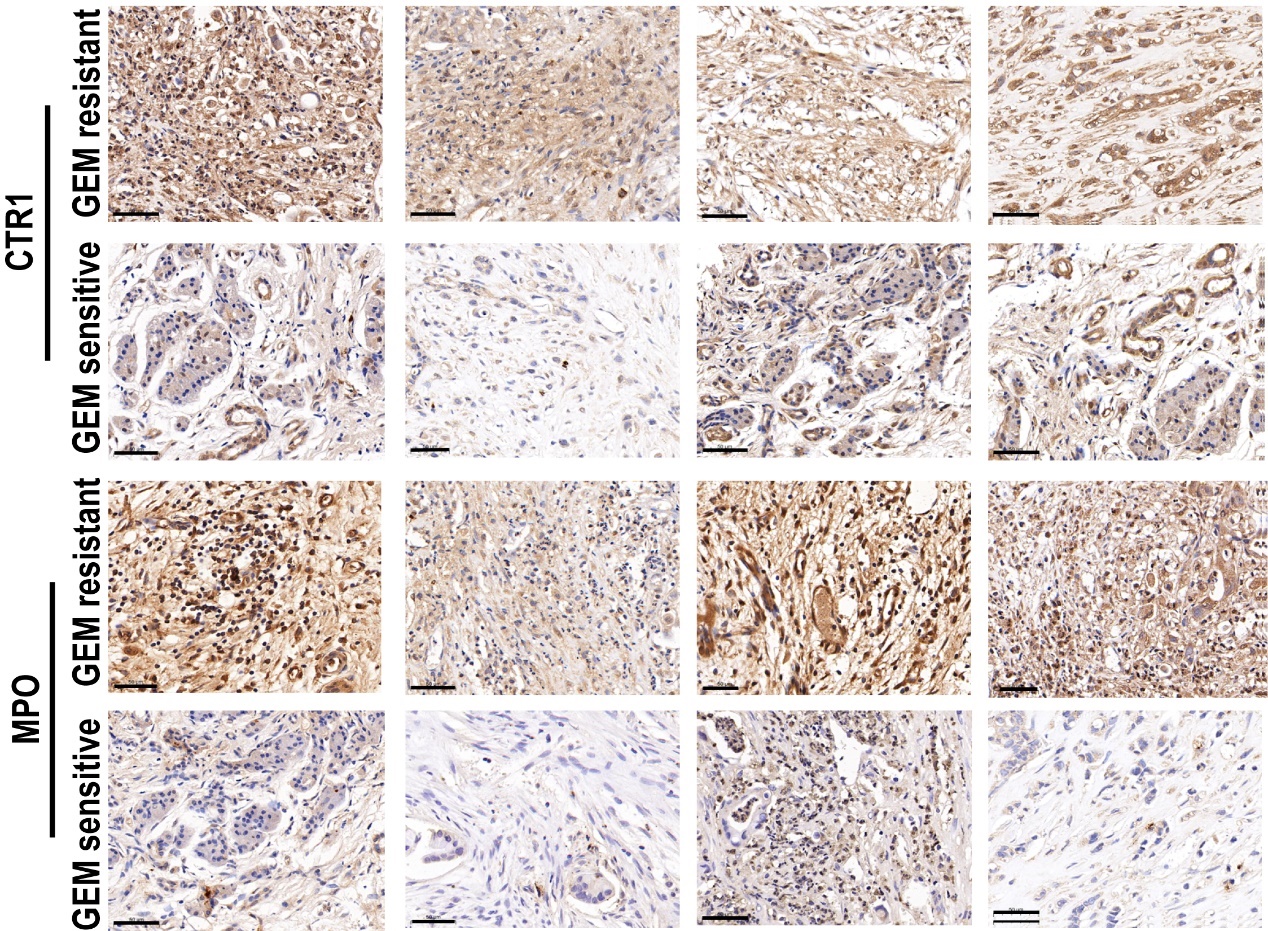
**

**Supplementary Figure 7.**Immunohistochemical (IHC) validate the expression of CTR1 and MPO in gemcitabine-resistant versus sensitive PDAC specimens (n=4).
