# Supplementary material for: Blockage of cuproplasia inhibits pancreatic tumour-associated neutrophils infiltration through TRAF6/STAT3/CCL2 pathway
Source: Br J Cancer. 2026 Apr 14;135(1):17–32. doi: 10.1038/s41416-026-03371-8 (PMC13269755; doi:10.1038/s41416-026-03371-8)
Supplement: Supplementary file 2 — Supplementary table 1 [file 41416_2026_3371_MOESM2_ESM.docx]

| Primer sequences |
| --- |
| CTR1 F: 5’-TTGGCTTTAAGAATGTGGACCT-3’ |
| CTR1 R: 5’-CATAAGGATGGTTCCATTTGGT-3’  CCL-2 F: 5’-CAGCCAGATGCAATCAATGCC-3’  CCL-2 R: 5’-TGGAATCCTGAACCCACTTCT-3’ |
| β-Actin F: 5’-CATGTACGTTGCTATCCAGGC-3’ |
| β-Actin R: 5’-CTCCTTAATGTCACGCACGAT-3’ |

Supplementary table 1.The primers of CTR1, CCL2 and β-Actin(negative control).
