# Supplementary material for: Blockage of cuproplasia inhibits pancreatic tumour-associated neutrophils infiltration through TRAF6/STAT3/CCL2 pathway
Source: Br J Cancer. 2026 Apr 14;135(1):17–32. doi: 10.1038/s41416-026-03371-8 (PMC13269755; doi:10.1038/s41416-026-03371-8)
Supplement: Supplementary file 3 — Supplementary table 2 [file 41416_2026_3371_MOESM3_ESM.docx]

Supplementary table 2. List of antibodies.

| Antibody | Cat.No | Manufacturer | | Location |
| --- | --- | --- | --- | --- |
| anti-GAPDH | 60004-1-Ig | Proteintech | Wuhan, China | |
| anti-CTR1 | ab129067 | Abcam | Cambridge, UK | |
| anti-CK19 | ET1601-6 | HUABIO | Hangzhou, China | |
| anti-CD31 | ER31219 | HUABIO | Hangzhou, China | |
| anti-α-SMA | ET1607-53 | HUABIO | Hangzhou, China | |
| anti-MPO | HA601249 | HUABIO | Hangzhou, China | |
| anti-TRAF6 | R1311-2 | HUABIO | Hangzhou, China | |
| anti-JAK2 | 3230T | Cell Signaling Technology | Massachusetts, USA | |
| anti-p-JAK2(Tyr1007) | 4406T | Cell Signaling Technology | Massachusetts, USA | |
| anti-STAT3 | 9139T | Cell Signaling Technology | Massachusetts, USA | |
| anti-p-STAT3(Y705) | ab267373 | Abcam | Cambridge, UK | |
| anti-Actin | 66009-1-Ig | Proteintech | Wuhan, China | |
| anti-CCL2 | 26161-1-AP | Proteintech | Wuhan, China | |
| anti-CD11b | 561114 | BD | NJ, USA | |
| anti-LY6G | 18359S | BD | NJ, USA | |
| anti-CCR2 | 130-117-548 | BD | NJ, USA | |
| anti-CD3e | 561826 | BD | NJ, USA | |
| anti-CD8a | 561109 | BD | NJ, USA | |
